# Self-Sampling for SARS-CoV-2 Diagnostic Testing by Using Nasal and Saliva Specimens: Protocol for Usability and Clinical Evaluation

**DOI:** 10.2196/24811

**Published:** 2021-05-28

**Authors:** Mohammed Majam, Vanessa Msolomba, Lesley Scott, Wendy Stevens, Fadzai Marange, Trish Kahamba, Francois Venter, Donaldson Fadael Conserve

**Affiliations:** 1 Ezintsha, Wits Health Consortium University of the Witwatersrand Johannesburg South Africa; 2 Department of Molecular Medicine and Haematology University of the Witwatersrand Johannesburg South Africa; 3 Department of Prevention and Community Health Milken Institute School of Public Health The George Washington University Washington, DC United States

**Keywords:** SARS-CoV-2, SARS-CoV-2SS, testing, COVID-19, South Africa, usabillity, self-sampling, diagnostic, sensitivity, specificity

## Abstract

**Background:**

SARS-CoV-2 is a novel coronavirus discovered in December 2019 and is currently the cause of the global COVID-19 pandemic. A critical aspect of fighting this pandemic is to obtain accurate and timely test results so that patients who have tested positive for COVID-19 can be identified and isolated to reduce the spread of the virus. Research has shown that saliva is a promising candidate for SARS-CoV-2 diagnostics because its collection is minimally invasive and can be reliably self-administered. However, little research has been conducted on saliva testing and SARS-CoV-2 self-sampling (SARS-CoV-2SS) in Sub-Saharan Africa.

**Objective:**

The primary objective of this study is to comparatively evaluate the clinical sensitivity and specificity of nasal and oral samples self-collected by individuals for SARS-CoV-2 testing against a reference method involving sample collection and testing by a health care professional. The secondary objectives of this study are to evaluate the usability of nasal self-sampling and saliva self-sampling as a sample collection method for SARS-CoV-2 diagnostic testing by using failure mode and error assessment.

**Methods:**

Participants will be recruited from the general population by using various methods, Participants will be screened progressively as they present at the clinical trial sites as well as in primary health care catchment areas in the inner city of Johannesburg, South Africa. In the event that recruitment numbers are low, we will use a mobile van to recruit participants from outlying areas of Johannesburg. We aim to enroll 250 participants into this study in approximately 6 weeks. Two sample types—a self-administered nasal swab and a self-administered saliva sample—will be collected from each participant, and a health care professional will collect a third sample by using a nasopharyngeal swab (ie, the standard reference method).

**Results:**

This protocol has been approved by the University of the Witwatersrand Human Research Ethics Committee on July 31, 2020 (Protocol number EzCov003). As of May 13, 2021, 120 participants have been enrolled into the study.

**Conclusions:**

SARS-CoV-2SS may offer many benefits to individuals, by allowing for initial self-identification of symptoms and collection of samples without involving third parties and potential risk of infection provided the sample can be safely processed via a collection system. The results of this study will provide preliminary data on the acceptability, feasibility, and usability of SARS-CoV-2SS among the general population for its future implementation.

**International Registered Report Identifier (IRRID):**

DERR1-10.2196/24811

## Introduction

SARS-CoV-2 is a coronavirus novel to the human population that was discovered in December 2019 and is currently the cause of a global pandemic [1-3]. In South Africa, the first case of COVID-19, the disease caused by SARS-CoV-2, was confirmed on March 5, 2020. As of June 23, 2020, there were approximately 102,000 confirmed COVID-19 cases and 1991 deaths reported in South Africa [4]. The World Health Organization (WHO) declared COVID-19 as a public health emergency of international concern on January 30, 2020 [5] and, subsequently, declared it as a pandemic on March 11, 2020 [6]. The United States declared COVID-19 a national emergency on March 13, 2020 [7], and South Africa declared a national state of disaster on March 15, 2020 [8]. Despite the scale-up in screening and testing, accurate reporting of COVID-19 cases has been limited by the availability of diagnostic testing.

A critical aspect of fighting this pandemic is to obtain accurate and timely test results so that patients who have tested positive for COVID-19 can be identified and isolated to reduce the spread of the virus. The virus is known to spread via both symptomatic and asymptomatic infected individuals [9]; therefore, early detection of positive cases can considerably reduce the spread of the virus. Most testing methods currently in use for SARS-CoV-2 require viral genetic material isolated from nasal and throat swabs for reverse transcription–polymerase chain reaction (RT-PCR) assay [10]. The current standard testing procedures for COVID-19 require a trained health care worker (HCW) to collect throat samples from the patient (either via the nasal and/or oral cavity) [10]. This process requires the use of personal protective equipment (PPE), is uncomfortable for the patient, and places unnecessary strain on the health care system [11]. Oropharyngeal swabs are easier to collect (without training) than nasopharyngeal swabs [12]. Recent research has shown that the collection of nasal and/or midturbinate samples for SARS-CoV-2 PCR testing is as effective as nasopharyngeal specimen collection [13]. This evidence therefore allows one to explore the options of self-sampling. One study conducted in the United States [13] shows the clinical usefulness of tongue, nasal, or midturbinate samples collected by patients as compared with nasopharyngeal samples collected by HCWs for COVID-19 diagnosis.

Adoption of techniques for sampling by patients can reduce PPE use and provide a more comfortable patient experience. Studies have also shown that saliva is a promising candidate for SARS-CoV-2 diagnostics because its collection is minimally invasive and can be reliably self-administered. Moreover, saliva has shown comparable sensitivity to nasopharyngeal swabs in the detection of other respiratory pathogens, including endemic human coronaviruses [14,15]. However, little research has been conducted on saliva testing and self-sampling for COVID-19 in Sub-Saharan Africa. In the proposed study, we will conduct validation of the use of self-administered nasal swab and saliva collection for SARS-CoV-2 detection to inform the implementation of COVID-19 self-sampling and, eventually, COVID-19 self-testing in South Africa. The primary objective of this study is to comparatively evaluate the clinical sensitivity and specificity of nasal and oral samples self-collected by individuals for COVID-19 PCR testing against the reference method (ie, nasopharyngeal swab) involving sample collection and testing by an HCW, which is the method currently used by the central laboratory for testing of symptomatic patients with COVID-19 symptom-onset in ≤7 days. The secondary objectives of this study are to evaluate the usability of nasal and saliva self-sampling as a sample collection method for COVID-19 diagnostic testing by using failure mode and error assessment.

## Methods

### Study Participants

Study participants will be recruited from the general population using the following inclusion criteria: (1) individuals aged 18 years and older; (2) individuals willing to provide consent; and (3) individuals reporting symptoms consistent with COVID-19, or those who have been in contact with a person diagnosed with COVID-19. The exclusion criteria are as follows: (1) individuals with active nose bleeds; (2) individuals with a previously confirmed COVID-19 RT-PCR test result; (3) individuals with facial injuries or trauma, or a condition that creates a mechanical barrier to safely collect clinical specimens; (4) individuals currently enrolled in a treatment study to evaluate an investigational drug and those who have started administering that drug; (5) individuals who have previously participated in this study; (6) individuals unable or unwilling to provide informed consent; (7) vulnerable individuals as deemed inappropriate for the study by the site principal investigator; (8) individuals who have undergone nasal specimen extraction (for any reason) within the last 24 hours (for these individuals, the specimen may be collected 24 hours after the standard-of-care sampling protocol); (9) personnel directly involved in the conduct of the study; and (10) individuals judged to be at significant risk of failing to comply with the provisions of the protocol so as to cause self-harm or seriously interfere with the validity of the study results.

Participants will be screened progressively as they present at the Ezintsha clinical trial sites in Johannesburg, South Africa, and neighboring areas. The study will also involve primary health care catchment areas in the inner city of Johannesburg. In the event that recruitment numbers are low, there is an option to utilize a mobile van to recruit participants from outlying areas of Johannesburg. A total of 250 participants will be enrolled into this study. Once participants have been identified through different recruitment channels, they will be approached and informed about the study and the role they will play in the study procedure. The objectives, rationale, eligibility requirements, and procedures of the study will be explained to the participants, while highlighting that their participation is purely voluntary. Risks and benefits of participation in the study and the rights of the participants will also be discussed.

### Sample Collection

Participants that meet the study’s inclusion criteria will be scheduled for an appointment and directed to the clinical research site. The eligible participants will be registered onto a biometric enrolment system. This system uses fingerprint scanning to eliminate the chance of duplicate enrolment or prior participation. Informed consent procedures will be performed electronically, when possible, before collection of demographics and other information related to occupation, place of work, and working environment (in terms of exposure to patients with COVID-19). From each participant from whom informed consent has been obtained, two sample types will be collected, that is, a self-administered nasal swab and a self-administered saliva sample.

Participants will receive the instructions for use along with the sample collection kits and proceed to collect their own nasal and saliva specimens. The sample collection kits will contain two dry swabs (one nylon flocked and one spun polyester swab), one universal container (for saliva collection), one barcoded requisition form, one rapid anti–COVID-19 antibody test (including an alcohol swab, a lancet, and a band aid), one dry blood spot (DBS) or plasma separation card (PSC) (also including an alcohol swab, a lancet, and band aid), an information sheet, a consent form to store DBS or PSC, and a barcode. An independent observer will observe the participants’ self-sample collection technique and note any deviations from the prescribed method in the instructions for use (see Multimedia Appendix 1). Participants will place their collected nasopharyngeal swab into a dry transport tube and their collected saliva sample container into the biohazard bag for transportation to the central laboratory. 

Once the participant has collected their own specimens, they will proceed to the designated HCW for collection of a nasopharyngeal, midturbinate, or oropharyngeal swab by a professional for the reference RT-PCR test for SARS-CoV-2. The HCW will insert the professionally collected specimen into a biohazard bag for transportation to the laboratory. A finger-prick rapid diagnostic test for COVID-19 antibody/antigen will then be performed by a research nurse. The same finger-prick site will be used for DBS collection for further serological testing. Briefly, for DBS collection, finger-prick blood sample will be collected using capillary tubes and loaded onto designated areas of the collection card. The cards will be left to dry for 4 hours at room temperature on a drying rack and then transported to the laboratory. After completing all study procedures, participants will receive reimbursement for their participation via an electronic fund transfer.

### Sample Processing

All swabs will be transported as dry swabs to the central laboratory for testing within 24 hours. Upon receipt at the laboratory, the swabs will be resuspended in phosphate buffer saline and maintained at 4°C until the time of testing. Saliva samples will be vortexed, pre-aliquoted into 1-ml volumes, and stored at –80°C until the time of testing; freeze-thaw cycles will be limited to one. These samples will be batched and processed at least once a week. Testing will be performed directly on raw saliva after mixing using a vortex and pipette. Qualitative detection of SARS-CoV-2 RNA will be performed on all collected swabs and saliva samples by real-time RT-PCR assay. DBS cards will also be stored at –80°C until the time of testing.

### Management of Participants to Limit Risk of SARS-CoV-2 Transmission

Since all participants will be at high risk of exposure to SARS-CoV-2 and potential infection, the following measures will be undertaken to avoid transmission risks. For any necessary physical interactions, participants and study personnel will be instructed to adhere to national and regional guidance for COVID-19–related safety measures. The study site will be organized in accordance with national and regional guidelines for limiting the spread of COVID-19, such that contact between participants with potential SARS-CoV-2 infection and other participants is restricted. The Ezintsha Research Centre is divided into designated zones. All study participants will be assessed in the sampling area, wherein strict measures will be enforced with all necessary PPE provided to the staff and participants. Contact between study participants and study personnel will be limited as far as possible. To reduce the burden on study participants, contact between study participants and study personnel will occur via telemedicine, text or direct messaging, or telephone as far as possible. Participants will be instructed to report any possible signs or symptoms of COVID-19 to the study personnel so that they can be considered as persons under investigation by the study team personnel, and they can be provided instructions on additional follow-up required during acute illness, if any.

### Management and Notification of Test Results

Upon completion of sample processing, all results will be reviewed and authorized by the laboratory technician. The Meditech Laboratory Information System will automatically deliver the results electronically to the health care facility. A paper copy of the results will be printed and delivered to the health care facility within 48 hours. The results of the professionally performed PCR test will be the only result that will be communicated to the participant via electronic means (eg, via WhatsApp, SMS, or direct call). The result of the self-sampled specimens, as well as any serology test, will only be used for validation purposes. The test results of these participants will be reported to the National Institute of Communicable Diseases, Notifiable Medical Conditions Surveillance system as per standard operating procedure.

### Digital Health Tool Validation

A critical component of self-sampling is the validation of an end-to-end digital pathway for the user. This will involve the creation of a technological algorithm that encompasses screening and risk identification, sample collection, and capturing of demographic information, as well as tracking through the laboratory system and subsequent reporting of results. Ezintsha, through its prior experiences with multiple projects, including but not limited to the HIV Self-Testing Africa (STAR) Initiative, has the requisite platforms off which such an integrated system can be built. Working through WhatsApp developers recognized by the Department of Health, Praekelt [16], we will develop an end-to-end digital pathway for COVID-19 self-sampling. This smartphone app will function as a workflow automation solution to enable remote monitoring of services across the clinical and laboratory value chain and provide a central data repository for program management.

### Data Analysis

The primary objective of this study is to estimate the sensitivity and specificity of participant-drawn self-samples and comparatively evaluate those against the reference method used for COVID-19 symptomatic patients (ie, those reporting onset of symptoms in ≤7 days). For the purpose of powering the study, the sensitivity of the self-samples is expected to be >80% at the lower limit of the two-sided 95% CI, whereas the specificity is expected to be >95% at the lower limit of the two-sided 95% CI compared to the reference method. The results of the self-sample matched pair of specimens (ie, nasal and saliva specimens) will be evaluated against the results of a validated PCR assay conducted at the central laboratory. No interim analyses will be performed during this trial. This statistical analysis plan will be developed and finalized before database lock and will describe the study population to be included in the analyses, in addition to the detailed analytical plans with endpoints and procedures for accounting for missing, unused, and spurious data. Demographic characteristics (ie, age, sex, and race) of each study group will be tabulated. The mean age (in addition to range and SD) by sex of the enrolled participants, as a whole and per group, will also be calculated. Given the study design and retention activities, measurable outcomes are expected for all participants. However, in the unlikely event of a missing test result, the missing data will be imputed.

## Results

This study protocol has been approved by the University of the Witwatersrand Human Research Ethics Committee on July 31, 2020 (Protocol number EzCov003). As of May 13, 2021, 120 participants have been enrolled into the study.

## Discussion

The WHO defines self-care as “the ability of individuals, families and communities to promote health, prevent disease, maintain health, and to cope with illness and disability with or without the support of a healthcare provider” [17]. Two major components of self-care are self-screening and self-sampling. Both strategies have been widely used and scaled-up in the last few years. Recently, HIV self-screening (more commonly referred to as HIV self-testing [HIVST]) has been used as a strategy to reach under-tested and key populations [18,19]. It is now firmly established globally that HIVST is acceptable and feasible with accurate performance and interpretation of results among diverse populations in the hands of *lay* or *untrained users* [20,21]. This modality has been allowed testing outside of conventional facilities, extending the reach of HIV programs within difficult-to-reach communities [18,22-24]. These studies have provided evidence on different distribution strategies, including web-based platforms, peers, sexual partners, and community health workers [23,25,26]. Similarly, these studies have assessed different approaches to verify HIVST results either via direct supervision by health care providers, requesting participants to return used HIVST kits; electronic transmission of photographs; or Bluetooth sensors [27]. These distribution and result verification strategies can be piloted with COVID-19 self-sampling and self-testing and potentially be used for integrating COVID-19 self-sampling and HIVST [28].

On April 21, 2020, the US Food and Drug Administration (FDA) provided an emergency authorization use for the first SARS-CoV-2 self-sampling (SARS-CoV-2SS) kit called Pixel by LabCorp [29]. More recently, the US FDA also provided an emergency authorization use for the first saliva-based in-home SARS-CoV-2 kits [30]. Lessons learnt through the phased approach used in HIVST may provide insights into how COVID-19 self-screening strategies may be adapted, built upon, and optimized [28]. SARS-CoV-2SS, if performed accurately, may offer many benefits to individuals, allowing for initial self-identification of symptoms and collection of samples without involving third parties (and a potential risk of infection) provided the sample can be safely processed via a collection system. A self-sampling or self-testing kit, containing a swab, instructions for sample collection, and packaging that allows for the safe isolation of the specimen could be constructed cost-effectively and, conceivably, be provided for use among high-risk HCWs, other essential service staff, and individuals who have been in close contact of people with COVID-19, as well as in hotspots (eg, in informal settlements where mass cases have been reported). These self-sampling kits can also be provided at pharmacies, for people to purchase, and at clinical laboratories, where people present themselves, thereby minimizing collection contact with the staff. Finally, should more widely available point-of-care diagnostics emerge, SARS-CoV-2SS may facilitate the sample collection process and reduce the overall turnaround time for receiving test results.

## Appendix

Multimedia Appendix 1 COVID-19 self-sampling: instructions for use.

## Abbreviations

DBS: dry blood pot
FDA: Food and Drug Administration
HCW: health care worker
HIVST: HIV self-testing
PPE: personal protective equipment
PSC: plasma separation card
RT-PCR: reverse transcription–polymerase chain reaction
SARS-CoV-2SS: severe acute respiratory syndrome coronavirus 2 self-sampling
STAR: Self-Testing Africa
WHO: World Health Organization
