# Supplementary material for: Self-Sampling for SARS-CoV-2 Diagnostic Testing by Using Nasal and Saliva Specimens: Protocol for Usability and Clinical Evaluation
Source: JMIR Res Protoc. 2021 May 28;10(5):e24811. doi: 10.2196/24811 (PMC8166267; doi:10.2196/24811)
Supplement: Multimedia Appendix 1 [file resprot_v10i5e24811_app1.pdf]

Ezintsha
INVESTIGATE INNOVATE ADVANCE

COVID-19 SELF SAMPLING: Instruction for use

NASAL SWAB

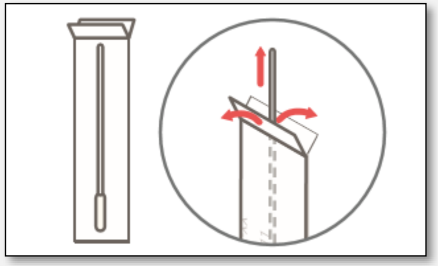

1. Open nasal swab

Remove the nasal swab from the wrapper by pulling the two ends of the wrapper apart (like you would open a band aid)

Be careful not to touch the tip

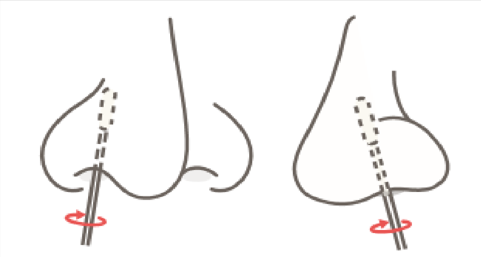

2. Swab nose

Gently insert the entire soft tip of the swab into the nostril until you feel a bit of resistance and rub it in a circle around your nostrils 4 times

Next, gently insert the same swab into the other nostril and rub it around the same way

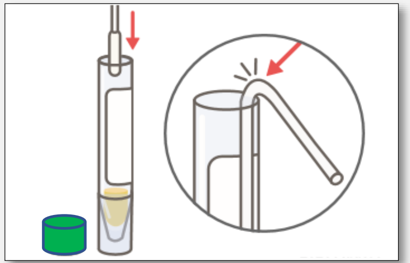

3. Put swab into tube

Lower the swab, tip first into the provided tube.

Screw the cap on tightly

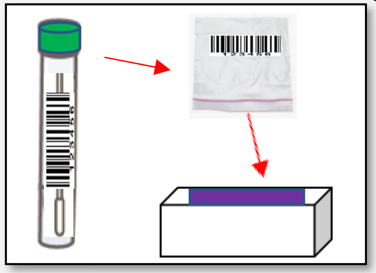

4. Package

Place tube into plastic bag provided and into kit box

SALIVA COLLECTION

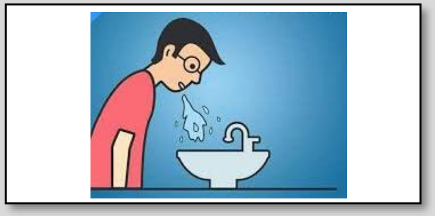

1. Rinse mouth

Rinse your mouth with water and discard.

Wait approximately 5 mins before collecting saliva

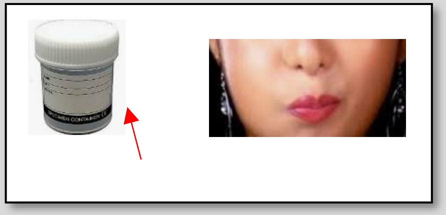

2. Prepare for collection

Remove the saliva collection container from the kit provided

Accumulate saliva in your mouth for about 30 seconds.

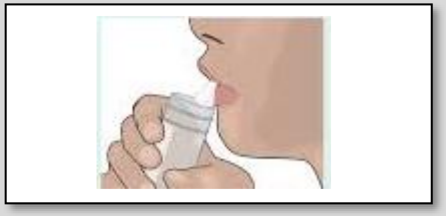

3. Collect Saliva

Open saliva collection container

Produce saliva and spit as much saliva into the container as possible, repeat the process to get approximately one tablespoon full

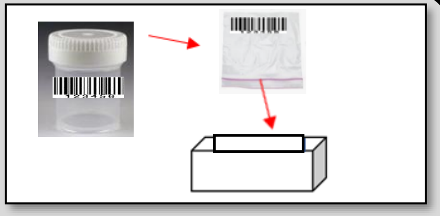

4. Package

Screw the cap on the container tightly and place back into the ziplock bag provided.

Place back into the kit box
